# Crystallisation in basaltic magmas revealed via *in situ* 4D synchrotron X-ray microtomography

**DOI:** 10.1038/s41598-018-26644-6

**Published:** 2018-05-30

**Authors:** M. Polacci, F. Arzilli, G. La Spina, N. Le Gall, B. Cai, M. E. Hartley, D. Di Genova, N. T. Vo, S. Nonni, R. C. Atwood, E. W. Llewellin, P. D. Lee, M. R. Burton

**Affiliations:** 1 School of Earth and Environmental Sciences, University of Manchester, Manchester, M13 9PL UK; 2 School of Materials, University of Manchester, Manchester, M13 9PL UK; 3 Research Complex at Harwell, Harwell Campus, OX 11 0FA, Didcot, UK; 4 Now at School of Metallurgy and Materials, University of Birmingham, Edgbaston, Birmingham, B15 2TT UK; 5 School of Earth Sciences, University of Bristol, Bristol, BS8 1RJ UK; 6 Diamond Light Source, Harwell Science and Innovation Campus, Didcot, OX11 0DE UK; 7 Present Address: UCL Mechanical Engineering, Torrington Place, London, WC1E 7JE UK; 8 Department Earth Sciences, Durham University, Durham, DH1 3LE UK

## Abstract

Magma crystallisation is a fundamental process driving eruptions and controlling the style of volcanic activity. Crystal nucleation delay, heterogeneous and homogeneous nucleation and crystal growth are all time-dependent processes, however, there is a paucity of real-time experimental data on crystal nucleation and growth kinetics, particularly at the beginning of crystallisation when conditions are far from equilibrium. Here, we reveal the first *in situ* 3D time-dependent observations of crystal nucleation and growth kinetics in a natural magma, reproducing the crystallisation occurring in real-time during a lava flow, by combining a bespoke high-temperature environmental cell with fast synchrotron X-ray microtomography. We find that both crystal nucleation and growth occur in pulses, with the first crystallisation wave producing a relatively low volume fraction of crystals and hence negligible influence on magma viscosity. This result explains why some lava flows cover kilometres in a few hours from eruption inception, highlighting the hazard posed by fast-moving lava flows. We use our observations to quantify disequilibrium crystallisation in basaltic magmas using an empirical model. Our results demonstrate the potential of *in situ* 3D time-dependent experiments and have fundamental implications for the rheological evolution of basaltic lava flows, aiding flow modelling, eruption forecasting and hazard management.

## Introduction

Magmas are multiphase mixtures consisting of liquid, crystals and bubbles. The arrangement and evolution of these textures during magma ascent or residence in a magma chamber reflects the physico-chemical history of the magma and thermodynamic properties of each component. Together, these textures control magma rheological behaviour and permeability for volatile flow (e.g., refs^1,2^). These, in turn, dictate magma crystallisation and volatile exsolution, providing powerful feedback mechanisms and complex non-linear behaviour. This richness makes volcanic processes fascinating, as there is a very wide range of possible behaviours with rapid transitions between styles of activity. However, these complex, interdependent processes must be accurately described if we are to succeed in producing accurate models of volcanic systems. Such models are the long-term goal for many volcanologists, as they will open the possibility of aiding volcano observatories in the interpretation of their observations, improving eruption forecasting and thereby allowing risk managers to keep populations safe whilst minimising costly evacuations.

The combined effects of crystal and vesicle textures on magma rheology and their relationship to eruptive style and intensity is a very active field of investigation. It has long been established that viscosity, the most important rheological property governing magma transport processes, is a function of magma composition, temperature, volatile content, crystal content, size, shape, distribution and orientation^2,3^ and vesicle content and arrangement^4,5^. Recently, earth scientists have shown that crystal shapes, besides their abundances, have a strong effect on magma rheological response^3,6^. It is for this reason that accurate knowledge of the processes determining the formation (e.g., nucleation, growth, phase changes) of crystal phases with time is of critical importance to realistically describe dynamic processes occurring during magma transport and syn- and post-eruptive emplacement.

Experimental work on crystallisation kinetics has been conducted through the study of 2D textures (e.g., refs^7–10^); however, the texture of a volcanic rock is the final product of a dynamic process and it is difficult to quantify with snapshot experiments and 2D measurements. This is particularly relevant when studying low-viscosity systems such as basalts where quench effects may alter the sample texture and chemistry. In addition, degassing and crystallisation are time-dependent processes that introduce strong non-linearity in magmatic and volcanic systems. Crystallisation occurs in two steps: nucleation and growth. Crystal growth is the process of the evolution of a single crystal nucleated in a melt with a regular structure into the characteristic arrangement of a crystalline Bravais lattice^11^. Growth could be related to a sequence of processes, from Ostwald ripening to crystal aggregation^12^ or dissolution, which significantly complicates the understanding of crystal textural evolution in both space and time. Although previous studies have attempted to investigate such processes on *ex situ* samples, as demonstrated in other systems^13,14^, only *in situ*, real-time quantification of 3D crystal sizes and morphologies can provide a realistic description of kinetics parameters such as crystal nucleation and growth rates, which will improve our knowledge of crystallisation and, consequently, magma rheological properties.

With a few notable exceptions, the volcanology community has conventionally assumed that the processes of magma degassing and crystallisation occur as an equilibrium response to depressurisation during magma ascent and eruption. However, it is now recognised that the timescales required to achieve equilibrium for both crystal growth^15–18^ and volatile exsolution^19–21^ are similar to, or longer than, ascent times for erupting basaltic magmas, and therefore disequilibria are expected to be ubiquitous in such systems. Neglecting disequilibrium crystallisation and degassing therefore limits quantitative modelling and our understanding of volcanic processes.

Disequilibrium processes are challenging to study because the *P, T*, volatile content, melt composition and rate-of-ascent parameter space is huge and because the traditional experimental techniques used to explore disequilibrium processes are challenging and time-consuming. Until now, the principle experimental approach has been 2D post-mortem examination of samples from laborious, conventional petrological experiments requiring interruption and quenching (e.g., refs^22,23^). By using a high-temperature mossainite cell, in ref.^12^ (and successive papers, see ref.^24^ and references therein) Schiavi *et al*. were able to optically observe *in situ* crystallisation of a basaltic andesitic melt at atmospheric pressure over time, but their results are limited to a 2D space and may be significantly influenced by surface effects.

In this study we report the first *in situ*, real-time 3D crystallisation experiments which constrain timescales of crystal kinetics in basaltic melts. We combine a high-temperature resistance furnace^14^ with the fast X-ray microtomographic capabilities of beamline I12 at the Diamond Light Source to directly quantify 4D (i.e. space and time) crystal nucleation and growth in a natural basaltic melt from Mt. Etna at atmospheric pressure. This allowed a full 3D picture with 3.2 micron^3^ voxel size to be captured every three minutes of the growing crystals, whose individual number and volumes could be quantified using post-processing segmentation techniques (see Methods for details).

Our experiments were intended to help inform part of the crystallisation process that occurs during lava emplacement. The Etna 1981 eruption produced a lava flow that extended up to 6 km in ~3 hours^25^, a similar timescale to our experiments. Whilst cooling would occur on the border of the flow, the core would have insufficient time to cool significantly in 3.5 hours, and so our isothermal experiments can capture the natural crystal dynamics. We cannot apply a shear force in these experiments, and therefore our results are only representative of the core of the lava flow where strain rate is minimal.

Cooling rates of basaltic lavas, measured at the surface and within active lava channels during emplacement at a variety of locations range from 0.01 to 15 °C/min^26,27^. The conditions investigated in the presented experiments, starting at super-liquidus temperature (1250 °C), imposing a single-step cooling and maintaining the temperature constant for 4 hours, likely represent the slowest cooling rates (~0.01 °C/min) of the interior of large lava flows and lava channels.

Our 4D experiments on basaltic melts at realistic magmatic temperatures are, to our knowledge, the first that have been conducted on natural basalts. We capture disequilibrium crystallisation pathways by investigating the size, shape, orientation and evolution of crystals as a function of both time and space. This powerful *in situ* experimental technique opens a new frontier in the research of complex multiphase magmas, which were previously limited to *ex situ* quench studies, which have major challenges in terms of repeatability and workload. Each experimental *in situ* 3D image we capture is the equivalent of a single *ex-situ* quench experiment, and during a four hour experiment we capture ~80 images. This is a step-change in how experimental petrology can be performed. The 4D X-ray microtomographic method represents therefore a paradigm shift in how experimental petrology and volcanological studies can be conducted.

## Results

### General observations

In our cooling crystallisation experiments (see Methods for details) a natural anhydrous basaltic melt sampled from the 2001 Mt. Etna eruption was heated at atmospheric pressure to 1250 °C on beamline I12 at the Diamond Light Source (Supplementary Table S1), and then cooled to 1150 °C or 1170 °C over a dwell time of 4 h (Supplementary Table S2). The experiments crystallised augitic clinopyroxene (Supplementary Fig. S1, Supplementary Table S3), hereafter referred to as simply pyroxene. We also observed abundant precipitation of Fe-Mg oxides during the entire duration of the experiments, generated primarily by exposure to the atmosphere, which produced uncontrolled redox conditions within our samples, favouring the nucleation of oxides. However, in this paper we focus primarily on pyroxene crystallisation. In fact, we could not perform a thorough quantitative image analysis on oxides owing to a fundamental resolution issue: a fraction of oxides is close to or below the resolution limit of our system (pixel size = 3.2 microns), producing artefacts and showing most of the oxides connected to each other and forming oxide clusters and/or chains (see Supplementary movie S2 in Methods). Indeed, their real spatial distribution consists of euhedral isolated oxide crystals, which are arranged in layers. Because of this resolution issue, it is very challenging to separate them and we could not reliably measure oxide number density, but we were instead able to quantify oxide volume fraction with time.

During the entire dwell time, full 3D datasets were acquired every 3 minutes for quantitative image analysis (Supplementary Table S4). In the following, we focus on crystal nucleation and growth kinetics results from an experiment cooling to 1150 °C (named ET1150 hereafter), and use an experiment cooling to 1170 °C (named ET1170 hereafter) to provide additional information on nucleation delay for pyroxenes and oxides.

MELTS calculations^28,29^ performed at ambient pressure and ambient fugacity (MH redox buffer), indicate that the oxide liquidus temperature is 1230 °C (which is the liquidus phase of the bulk composition), in agreement with Orlando *et al*. [ref.^30^]. The clinopyroxene liquidus temperature is 1188 °C for the composition of our starting material. This estimate is similar to experimental determinations of clinopyroxene liquidus temperatures from Etnean basalt compositions^16,30^. The single-step cooling method was adopted in order to induce basaltic melt crystallisation in response to an instantaneously applied thermodynamic driving force (i.e., undercooling, ∆*T* = *T*_liquidus_ − *T*_experimental_). On the basis of the calculated clinopyroxene liquidus temperature, the experiments were performed at nominal ∆*T* = 18 °C and 38 °C for ET1170 and ET1150 respectively. The oxide crystals were formed at nominal ∆*T* = 60 °C and 80 °C during experiments ET1170 and ET1150, respectively.

**Figure 1:**
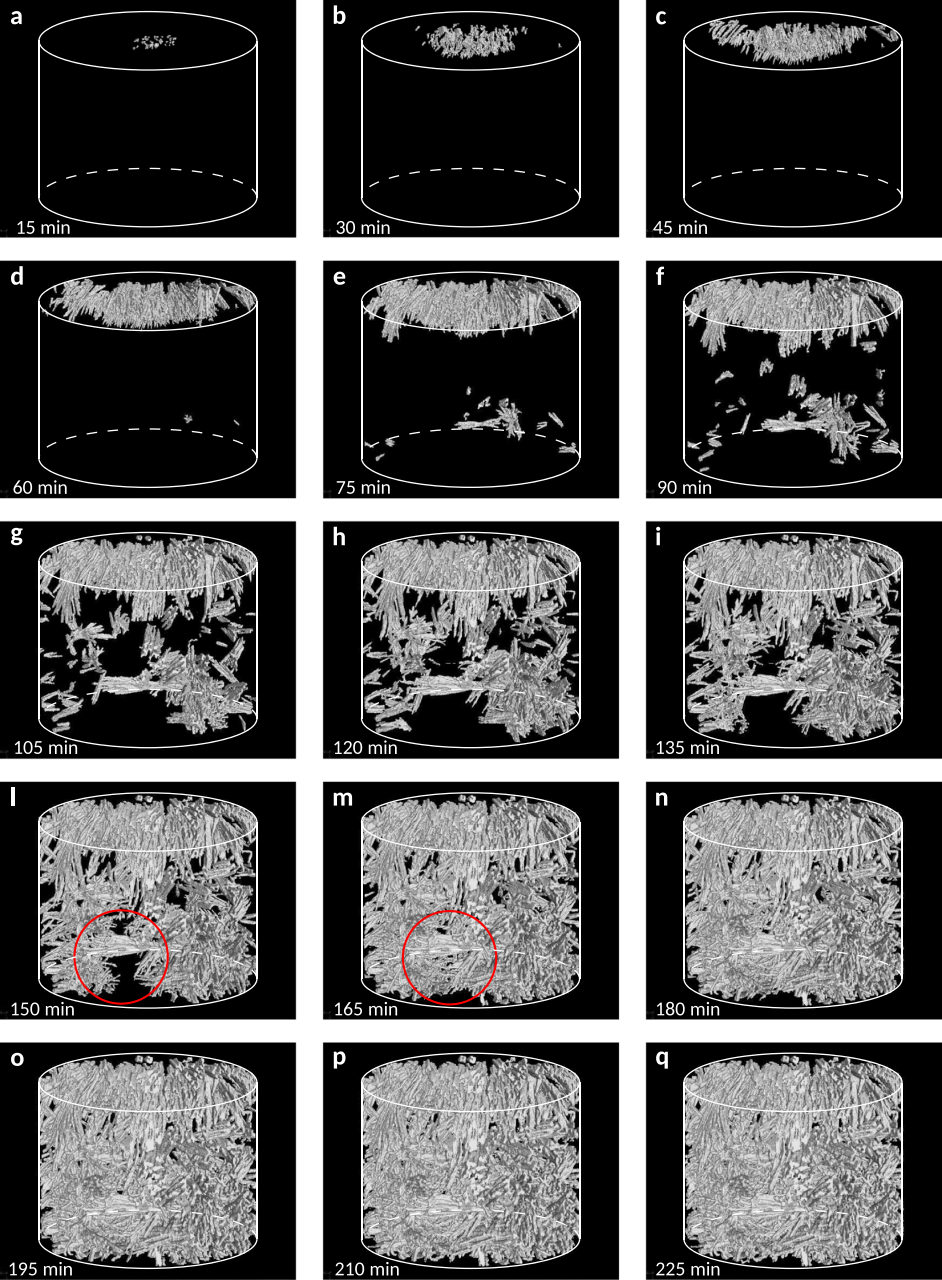
Crystallisation in a cooling basaltic melt at 1 atm. Volume renderings showing pyroxene crystallisation kinetics in single-step cooling experiment ET1150.Vertical field of view is 2 mm. Red circles in l) and m) indicate region of pyroxene crystal volume fraction increase in Fig. 2a. See text for further details.

We choose to focus on experiment ET1150, because experiment ET1170 crystallised only a very small volume fraction of pyroxene crystals (crystal volume fraction < 0.001) after 4 h at the melt/sample holder interface at the bottom of the sample, and ~0.11 ± 0.01 of oxide crystals. This implies that pyroxene crystallisation kinetics are very slow at ∆*T* = 18 °C, in agreement with ref.^16^. The nucleation, growth and textural development of pyroxene crystals with time is captured for the first time in Fig. 1 (see Supplementary Movie S1 for full experiment). Each frame illustrates successive crystal nucleation and/or growth, with a dwell time of 15 minutes between consecutive frames. Each frame represents a 3D volume rendering of the same representative digital segmented volume of interest (VOI) selected from the centre of each tomographic scan within experiment ET1150 for quantitative analysis (see Methods).

Pyroxene crystals in ET1150 are dominantly euhedral and their habits change from blocky to more elongate as crystallisation proceeds (Fig. 1). They are initially distributed as individual crystals (Fig. 1a–c); however, both crystal branching and crystal aggregates become ubiquitous as crystallisation continues (Fig. 1e,f and successive frames). Oxide crystals are spherical and mostly arranged in layers (see Methods, Supplementary Movie S2). In our 3D tomographic images, we did not find pyroxene crystals that nucleated from the bottom of our VOI. We observe what appears to be both heterogeneous and homogeneous nucleation. Pyroxene and oxide crystals nucleate heterogeneously from the top of the sample holder at the melt/air interface within the first 12 minutes (Fig. 1a,b, Table 1) and 6 minutes of the dwell time (Table 2), respectively. Because of our nominal resolution of 3.2 μm, we cannot however rule out the possibility that we are missing submicron crystals forming at the onset of dwell time, implying that the nucleation delay found in our experiments and reported in the following sections may be shorter. Heterogeneous nucleation at the melt/air and melt/sample holder interfaces represents the first nucleation event, although the latter becomes apparent in Fig. 1 only after 1 h of dwell time. The first pyroxene crystal nucleates in the melt after 60 minutes of dwell time (Fig. 1d). Because in the tomographic images we do not see any surface upon which this crystal could have nucleated, we assume that it nucleated homogeneously. We cannot however exclude that the nucleation of this first crystal in the bulk was heterogeneously promoted by a small pore or an oxide that cannot be resolved at the 3.2 μm pixel resolution used in our experiments^31^. This crystal nucleus promotes a spherulite-like crystal growth, consisting mostly of crystalline branches and crystal aggregates, visible near the bottom right-hand corner of our volume renderings (Fig. 1e–g and successive frames). After 60 minutes of dwell time crystallisation is dominated by heterogeneous nucleation events, generating branched polycrystalline morphologies.

**Table 1:** Experimental results of pyroxene crystallisation kinetics in single step-cooling experiment ET1150.

Frame# (time)	*ϕ*	*Nv* (mm^−3^)	*Iv* (mm^−3^ sec^−1^)	*Yv* (sec^−1^)	*Yiv* (sec^−1^)
5 (15 min)	4.3E-05 (2.6E-06)	4.27 (0.3)	4.70E-03 (3.3E-04)	4.75E-08 (3.30E-09)	4.75E-08
10 (30 min)	7.3E-04 (4.4E-05)	17.31 (1.2)	9.60E-03 (6.7E-04)	4.02E-07 (2.81E-08)	7.64E-07
15 (45 min)	2.3E-03 (1.4E-04)	28.04 (2.0)	1.00E-02 (7.0E-04)	8.51E-07 (6.0E-08)	1.74E-06
20 (60 min)	5.6E-03 (3.4E-04)	40.83 (2.8)	1.13E-02 (8.0E-04)	1.55E-06 (1.10E-08)	3.66E-06
25 (75 min)	0.011 (6.6E-04)	30.96 (2.2)	6.88E-03 (5.0E-04)	2.50E-06 (1.74E-07)	6.25E-06
30 (90 min)	0.016 (9.6E-04)	34.50 (2.4)	6.40E-03 (4.5E-04)	2.97E-06 (2.08E-07)	5.40E-06
35 (105 min)	0.021 (0.003)	49.98 (3.5)	7.93E-03 (5.5E-04)	3.31E-06 (2.32E-07)	5.36E-06
40 (120 min)	0.024 (0.001)	69.48 (4.9)	9.65E-03 (6.7E-04)	3.41E-06 (2.04E-07)	4.11E-06
45 (135 min)	0.025 (0.001)	84.11 (5.9)	1.04E-02 (7.3E-04)	3.12E-06 (2.18E-07)	7.51E-07
50 (150 min)	0.032 (0.002)	100.08 (7.0)	1.11E-02 (7.7E-04)	3.60E-06 (2.51E-07)	7.81E-06
55 (165 min)	0.053 (0.003)	90.08 (6.3)	9.10E-03 (6.4E-04)	5.30E-06 (3.70E-07)	2.32E-05
60 (180 min)	0.056 (0.003)	71.92 (5.0)	6.60E-03 (5.0E-04)	5.20E-06 (3.63E-07)	3.45E-06
65 (195 min)	0.065 (0.004)	78.01 (5.5)	6.70E-03 (5.0E-04)	5.60E-06 (3.92E-07)	1.04E-05
70 (210 min)	0.075 (0.004)	69.97 (4.9)	5.55E-03 (4.0E-04)	5.94E-06 (4.15E-07)	1.05E-05
75 (225 min)	0.079 (0.005)	71.67 (5.0)	5.31E-03 (4.0E-04)	5.84E-06 (4.10E-07)	4.51E-06
79 (237 min)	0.084 (0.005)	96.54 (6.7)	6.80E-03 (5.0E-04)	5.91E-06 (4.14E-07)	5.73E-06

*ϕ* = crystal volume fraction, *Nv* = Crystal number density, *Iv* = average crystal nucleation rate, *Yv* = average crystallisation rate, *Yiv* = instantaneous crystallisation rate. Values in parentheses are the standard deviation of the mean value.

**Table 2:** Experimental results of oxide crystallisation kinetics in single step-cooling experiment ET1150.

Frame# (time)	*ϕ*	*Yv* (sec^−1^)
5 (15 min)	0.03 (0.002)	3.33E-05 (2.31E-06)
10 (30 min)	0.04 (0.002)	2.22E-05 (1.55E-06)
15 (45 min)	0.06 (0.004)	2.22E-05 (1.55E-06)
20 (60 min)	0.08 (0.005)	2.22E-05 (1.55E-06)
25 (75 min)	0.09 (0.005)	2.00E-5 (1.40E-06)
30 (90 min)	0.10 (0.006)	1.85E-05 (1.30E-06)
35 (105 min)	0.10 (0.006)	1.60E-05 (1.11E-06)
40 (120 min)	0.14 (0.008)	1.94E-05 (1.4E-06)
45 (135 min)	0.14 (0.008)	1.73E-5 (1.21E-06)
50 (150 min)	0.16 (0.009)	1.77E-05 (1.24E-06)
55 (165 min)	0.16 (0.009)	1.62E-05 (1.13E-06)
60 (180 min)	0.16 (0.009)	1.50E-05 (1.04E-06)
65 (195 min)	0.16 (0.009)	1.40E-05 (9.60E-07)
70 (210 min)	0.17 (0.01)	1.35E-05 (9.44E-07)
75 (225 min)	0.17 (0.01)	1.26E-05 (8.82E-07)
79 (237 min)	0.17 (0.01)	1.20E-05 (8.40E-07)

*ϕ* = crystal volume fraction, *Yv* = average crystallisation rate.

Values in parentheses are the standard deviation of the mean value.

It is important to highlight that, in this study, the total crystal volume in each 3D frame reflects a combination of both nucleation and crystal growth, and is the sum of the volume of individual crystals, crystal branches and crystal aggregates. The calculated crystal number density with time also includes all types of crystal morphologies (individual, branched and aggregates). For this reason, in the following discussion we use the term *crystallisation rate* (expressed as crystal volume fraction over time), which more faithfully illustrates our combined nucleation and growth processes, rather than using *crystal growth rate*, which conventionally relates only to individual crystal growth^7,32^. Specifically, we use *average crystallisation rate* to describe the crystallisation rate averaged over the entire crystal nucleation and growth period, and *instantaneous crystallisation rate* to describe the crystallisation rate between two successive temporal frames (see Methods for further details).

### Pyroxene and oxide crystal volume fraction and crystallisation rate

**Figure 2:**
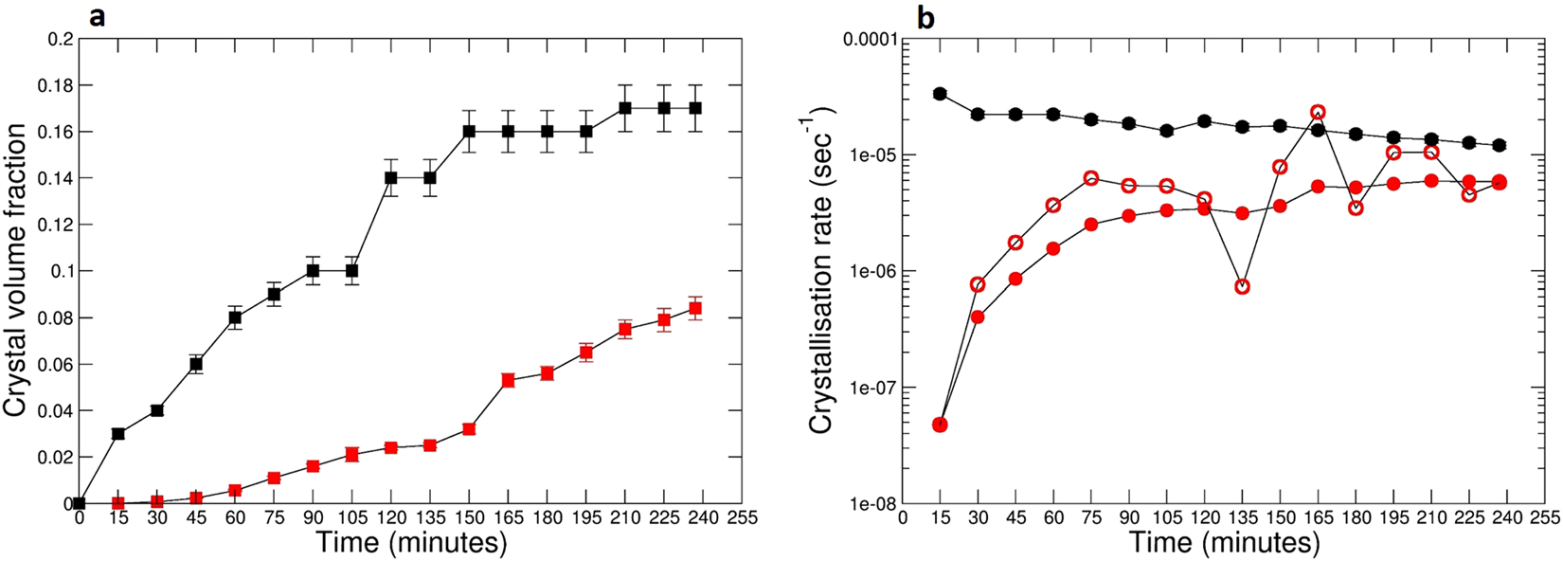
Crystal volume fraction and crystallisation rate in single-step cooling experiment ET1150 with time. Pyroxene (red squares) and oxide (black squares) crystal volume fraction (**a**), pyroxene average (closed red circles) and instantaneous (open red circles) crystallisation rate, and oxide average crystallisation rate (closed black circles) (**b**). See text for further details.

During experiment ET1150 pyroxene crystal volume fraction varies between 4.3E-05 after the first 15 minutes of dwell time and 0.084 at the end of the experiment (Fig. 2a, Table 1). Pyroxene crystal volume fraction grows slowly in the first 60 minutes of dwell time; it then increases almost linearly until the end of the experiment (Fig. 2a). We observe a rapid increase in the volume fraction, from 0.032 to 0.053 (see red circles in Fig. 1l,m), between 150 minutes and 165 minutes of the dwell time. Pyroxene average crystallisation rate displays values from 4.75E-08 sec^−1^ to 5.94E-06 sec^−1^ (Fig. 2b). As a first order approximation, the average crystallisation rate (Fig. 2b) increases rapidly in the first hour (up to 2E-06 sec^−1^), plateauing to an almost constant rate in the latter part of the experiment (from 3E-06 sec^−1^ to 6E-06 sec^−1^), meaning that equilibrium conditions are not reached. The instantaneous crystallisation rate shows, in the first hour, a similar behaviour to the average crystallisation rate, increasing up to 4E-06 sec^−1^ (see Fig. 2b). After the first hour, although the average crystallisation shows an almost constant rate, the instantaneous crystallisation displays several fluctuations ranging from 7E-07 sec^−1^ to 3E-05 sec^−1^, implying that the crystallisation rate is not constant with time as it might appear from a first order approximation.

The crystal volume fraction of Fe-Mg oxides varies between 0.03 after the first 15 minutes of dwell time and 0.17, the latter values reached at the end of the 4 h dwell time in experiment ET1150 (Fig. 2a, Table 2). Oxide crystal volume fraction grows quickly in the first 150 minutes of dwell time, then plateauing in the latter part of the experiment (Fig. 2a). Oxide average crystallisation rate is the highest at the beginning of dwell time (Fig. 2b, Table 2), and it then decreases very slowly from 3.33E-05 sec^−1^ to 1.20E-05 sec^−1^ until the end of the experiment.

### Pyroxene and oxide crystal nucleation delay, pyroxene number density and nucleation rate

**Figure 3:**
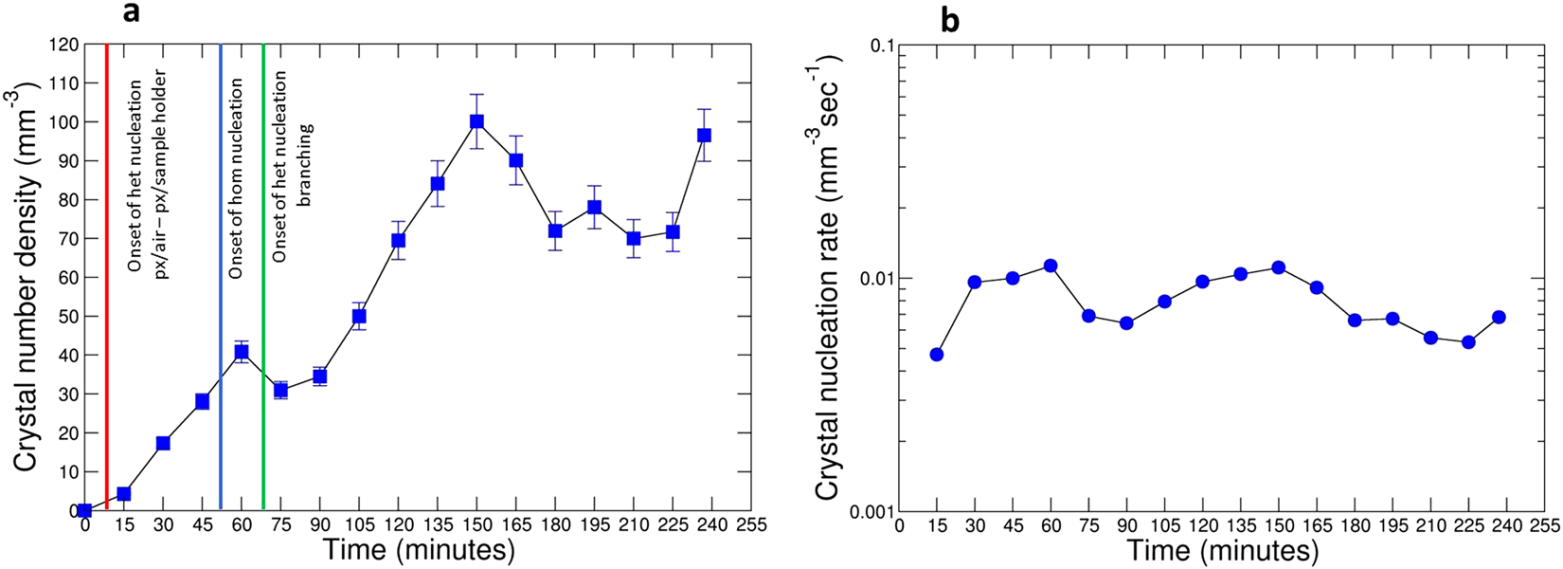
Crystal number density and crystal nucleation rate in single-step cooling experiment ET1150 with time. Pyroxene crystal number density (**a**) and pyroxene average crystal nucleation rate (**b**). Red, blue and green lines in (**a**) indicate onset of heterogeneous nucleation, homogeneous nucleation and heterogeneous nucleation branching. See text for further details.

The nucleation delay for heterogeneous pyroxene and oxide nucleation is, respectively, within 12 minutes and 6 minutes for experiment ET1150 (∆*T*_*Px*_ = 38 °C; ∆*T*_*Ox*_ = 80 °C), and 30 minutes and 12 minutes for experiment ET1170 (∆*T*_*Px*_ = 18 °C; ∆*T*_*Ox*_ = 60 °C), demonstrating that the nucleation delay is inversely proportional to ∆*T*. This is consistent with the nucleation behaviour of plagioclase observed during isothermal decompression experiments^10^, and of olivine in cooled basaltic melts at ambient pressure^33^. These results suggest that nucleation delay for silicate and oxide crystals in basaltic melts is strongly controlled by ∆*T*, whether crystallisation is induced by ambient cooling or isothermal decompression. The pyroxene crystal number density ranges between about 4 and 100 mm^−3^ over the course of experiment ET1150 (Fig. 3a, Table 1). We observe three clear spikes in the crystal number density as time progresses: the first and second occur after 60 minutes and 150 minutes in the dwell time, while the third occurs at the end of the experiment (Fig. 3a). These spikes suggest that pyroxene nucleation occurs in pulses. Pyroxene average crystal nucleation rates range between 4.70E-03 mm^−3^ sec^−1^ and 1.13E-02 mm^−3^ sec^−1^ (Fig. 3b, Table 1). Owing to the choice of VOI, our crystal nucleation results are challenging to interpret. Pyroxene crystals nucleated heterogeneously at the melt/sample holder interface within 12 minutes of the dwell time, at the same time as pyroxene crystals nucleated at the melt/air interface. However, the former were initially not included in our VOI. In fact, in the choice of the VOI, we excluded regions in the proximity of the sample holder walls (Supplementary Fig. 2, see Methods), where crystal segmentation was highly subjective owing to sample surface roughness and the presence of many highly phase-contrasted small bubbles. Therefore, pyroxene crystals at the melt/sample holder interface entered and were counted in our selected VOI only after 1 h of the dwell time, with a delay of 45 minutes after they nucleated, implying that the nucleation trend that we observe from 1 h onwards could be potentially shifted backwards in time.

## Discussion

Our 4D *in situ* synchrotron X-ray tomographic results capture for the first time the kinetics of pyroxene nucleation and growth and of oxide crystallisation, revealing distinct stages of the process quantitatively. Past experiments on crystal nucleation and growth have mostly focussed on the study of feldspars in rhyolitic and basaltic melts (e.g., refs^10,34^). Studies on pyroxenes are very few and are limited to conventional petrological experiments longer than 12 h^9^. Oxide crystallisation kinetics are also poorly experimentally investigated^3,30^, however, they could have an important role in heterogeneous nucleation of pyroxene in basaltic melts promoting, for example, crystal aggregation^35^. Because longer experimental durations provide lower apparent kinetic rates, previous studies may therefore have underestimated pyroxene nucleation and growth rates. This has been already highlighted, for example, in decompression-induced crystallisation experiments of a hydrous basaltic melt^10^, where they found that plagioclase nucleation and growth rates estimated in their 1 to 8 h experiments are up to one order of magnitude higher than those of plagioclase obtained from longer duration studies^9^. Recently, shorter *in situ* temporal observations of pyroxene crystallisation in a high-K basaltic melt were optically conducted at atmospheric pressure with a high-temperature moissanite cell^24^, but only 2D surface measurements were performed, which might prevent identifying textural features related to crystal nucleation and growth kinetics that are instead apparent in 3D (e.g., growth in pulses etc.). Our experiments uniquely measure the complex 3D crystal geometries *in situ*, allowing the first quantitative investigation of crystallisation kinetics in a natural magma as a function of time. For these reasons, the only kinetic data that can reliably be compared with our results are those measured in 3D trachytic feldspar spherulites^36^. In ref.^36^, Arzilli *et al*. report feldspar average crystallisation rates between 1E-07 sec^−1^ and 1E-08 sec^−1^ in cooling crystallisation experiments of trachytic melts lasting 4 and 6 h. These crystallisation rates are up to one order of magnitude lower than rates obtained for our pyroxene crystals (1E-06 sec^−1^/1E-08 sec^−1^, see Fig. 2b and Table 1), suggesting that the kinetics of nucleation and crystal growth are potentially faster in basaltic than trachytic melts owing to their lower viscosity^37^.

In experiment ET1150 we observe three distinct pyroxene nucleation events. The first heterogeneous nucleation event occurs at the melt/air and melt/sample holder interfaces and starts just a few minutes after the beginning of the dwell time (Figs 1a and 3a). When magma reached sub-liquidus conditions, crystal nucleation was firstly favoured by the presence of pre-existing surfaces such as melt/air and melt/sample holder interfaces^38^, which will be cooler and may have very small oxides (<3.2 µm) that act as heterogeneous nuclei, although the latter cannot be resolved at the resolution used in our experiments. Bubble surfaces (melt/gas interfaces) in a magma can induce heterogeneous crystal nucleation^36,39–41^. Our results therefore suggest that nucleation of pyroxene may be faster in the presence of bubbles, which has important implications for conduit dynamics during magma degassing. Because magma rheology is profoundly affected by the presence of crystals, bubble enhanced crystal nucleation will increase magma viscosity, which, in turn, affects the overall magma fluid dynamics in the conduit^42^. Finally, it is important to highlight that the initial oxide crystallisation may affect the initial pyroxene crystal nucleation but it does not affect pyroxene crystal growth kinetics as the two phases appear to follow distinct crystallisation trends (Fig. 2).

We observe that the first crystal (termed homogeneous) nucleated in the bulk after 1 h initiated the generation of the large spherulite-like texture, mostly consisting of crystal aggregates and branched polycrystalline pyroxenes. From Fig. 1e onwards, it is apparent that homogeneously nucleated pyroxene crystals and pyroxene crystals nucleated on pre-existing surfaces start to develop polycrystalline morphologies by branching. This implies that a second type of heterogeneous nucleation occurred on pre-existing pyroxene crystals, i.e. at solid/solid interfaces. This type of nucleation dominates from 75 minutes to the end of the dwell time, while homogeneous nucleation and growth of individual crystals are secondary processes during this time interval. Indeed, when the system is far from equilibrium, the pre-existing crystal surface can be perturbed by heterogeneities and instabilities, resulting in the nucleation of new grains at the growth front^36,43,44^, and yielding a rich variety of polycrystalline growth patterns. Heterogeneous nucleation leads to a decrease in the interfacial surface energy, reducing the work of cluster formation, *W*. The work of cluster formation on a pre-existing surface can be defined by^45^:

1 $${W}_{{\rm{het}}}=1/4\,{W}_{{\rm{\hom }}}(2-3cos{\rm{\theta }}+co{s}^{3}{\rm{\theta }})$$

where θ is the dihedral angle at the contact between two crystals and the melt. The dihedral angle (θ) is related to the ratio of interfacial free energies (solid-liquid interfacial energy/solid-solid grain boundary energy)^45^ expressed as:

2 $$\frac{{\sigma }_{sl}}{{\sigma }_{ss}}=\frac{1}{2cos\frac{\theta }{2}}$$

where *σ*_*sl*_ and *σ*_*ss*_ are the solid-liquid and solid-solid interfacial free energies, respectively. For low θ, the interfacial energy ratio will be low and heterogeneous nucleation will be energetically favoured^38,46^. In this study, dihedral angles (θ) between a pre-existing crystal and the new grain were measured, using the Avizo 3D software (FEI Visualization Sciences Group), as indicators of the interfacial energy ratio. Dihedral angles range between 30° and 54°, implying that heterogeneous nucleation on pre-existing crystals was strongly promoted in comparison with homogenous nucleation. Finally, we find that pyroxene nucleation and crystallisation rates (Figs 2b and 3b) in experiment ET1150 differ from those that would be predicted by either the classical crystal size distribution theory^47–49^ or successive revisions of this theory (see, for example, ref.^24^). The former states that crystal nucleation and growth occur simultaneously over a large temperature and time interval at a constant rate, while the latter predicts instead that the nucleation of each crystal phase is limited to a short event, followed by a long period of crystal growth and annealing. Our data on pyroxene crystals demonstrate the occurrence of at least three nucleation events over a period of 4 h (Fig. 3a). In addition, the evolution of instantaneous crystallisation allows us to identify three growth-dominated crystallisation events at 75, 165 min and at the end of the experiment (Fig. 2b), highlighting that crystallisation is not constant but proceeds through pulses over time.

Multiple crystal nucleation events have been ascribed by Armienti *et al*. (1994) to sudden temperature and pressure perturbations during magma ascent and storage^50^. We believe instead that our multiple pyroxene crystal nucleation events and growth through pulses at high temperature and atmospheric pressure are likely to reflect different interfacial free energies and delays in nucleation under disequilibrium conditions.

**Figure 4:**
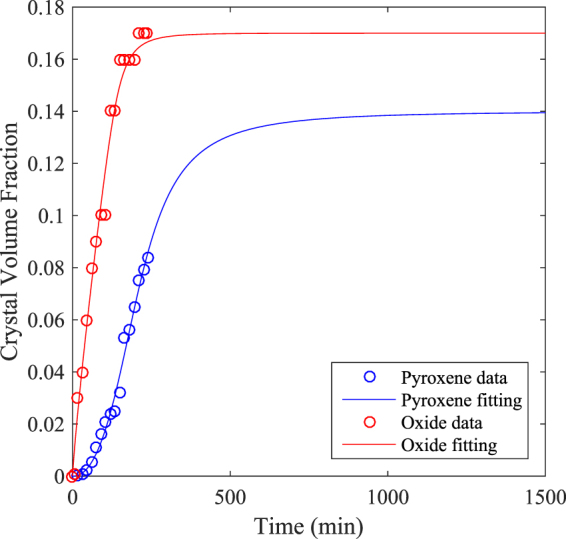
Disequilibrium crystallisation pathway in single-step cooling experiment ET1150 with time. Pyroxene (in blue) and oxide (in red) model of the disequilibrium crystallisation pathway obtained from the fitting of the experimental data. See text for further details.

Experiment ET1150 displays a pyroxene crystallisation trend (Fig. 2a,b) that differs from that assumed by numerical models of magma crystallisation in volcanic conduits. We find that pyroxene volume fraction increases slowly in the first 60 minutes, then almost linearly over the remaining 3 h of the experiment (Fig. 2a). The model used by La Spina *et al*. in refs^18,51^ instead assumes the opposite trend, where the crystal volume fraction increases rapidly at the onset of crystallisation and slows with time. In order to derive a new formulation for disequilibrium crystallisation in basaltic magmas, we fitted our empirical data on crystal volume fraction of pyroxene versus time. In our experiments, however, the equilibrium crystal volume content of pyroxene is not achieved; therefore we cannot describe the complete pathway up to the equilibrium value. We have calculated this number using MELTS^28,29^. We also assumed that at a certain point, as soon as the actual crystal content is very close to the equilibrium value, the crystallisation rate should decrease with time. However, since in our experiment we do not reach the equilibrium value for pyroxene, we cannot constrain exactly when the crystallisation rate starts to decrease to zero. With these assumptions, we found that the best fitting for the disequilibrium crystallisation in our experiment can be obtained from:

3 $${{\Phi }}_{c}(t)={{\Phi }}_{c,0}+({{\Phi }}_{c}^{eq}-{{\Phi }}_{c,0}){[arctan(\frac{\pi }{2}{c}_{1}{t}^{{c}_{2}})]}^{{c}_{3}},$$

where ${{\Phi }}_{c,0}$ is the initial crystal volume fraction, ${{\Phi }}_{c}^{eq}$ is the equilibrium value, ${{\Phi }}_{c}(t)$ is the actual value at the time *t*, and *c*_1_,*c*_2_ and *c*_3_ are proper fitting parameters. Given an initial crystal volume fraction of 0 and the equilibrium value of 0.14 (obtained from MELTS calculations), the values of the fitting parameters *c*_1_,*c*_2_ and *c*_3_ for the pyroxene crystal volume fraction curve are 9.46e-7, 2.57 and 0.86 respectively (Fig. 4, blue line).

Using the same equation, we were able to fit also the oxide crystal volume fractions with time. Compared to pyroxene, we do not need to estimate the equilibrium oxide content, since it reaches the equilibrium value during the course of the experiment (i.e. 0.17). The red line in Fig. 4 shows the fitting curve of the oxide crystal volume fraction obtained assuming the fitting parameters *c*_1_,*c*_2_ and *c*_3_ equal to 5.4e-9, 3.84 and 0.22, respectively.

Equation (3) is expressed with a parametric formulation, and therefore it can be applied to describe disequilibrium crystallisation pathways of different crystal phases, in different conditions and for different natural systems. Clearly, different conditions such as pressure, temperature, oxygen fugacity, etc., will affect the fitting parameters. However, the overall trend of the disequilibrium crystallisation will remain similar to that found in this study, suggesting that Eq. (3) will be valid also for different conditions.

In addition, Fig. 4 illustrates the comparison between the empirical crystal volume fractions obtained from our experiment and the fitting curves obtained from Eq. (3). Although the oxide crystal content seems to reach the equilibrium value within 240 minutes (4 h), using our equation, we determine that the difference between the equilibrium value of pyroxene and the actual value becomes small after 1000 minutes (~17 h), and it reaches equilibrium after ~1500 minutes (~25 h). The trend of pyroxene illustrated by Fig. 4 is similar to that obtained by Melnik and Sparks in ref.^52^, where they model the evolution of crystal content in the Montserrat magma over time. In their Fig. 2, they show that the crystal content initially increases slowly and then grows linearly until it eventually reaches equilibrium. This finding has implications for magma rheology and dynamics in volcanic conduits and lava flows, whereby slower crystallisation implies a lower magma viscosity and a faster magma flow rate. Our results strongly suggest that disequilibrium crystallisation processes in basaltic systems may be more pronounced than has previously been predicted.

The results of this study are important to quantify the crystallisation kinetics of basaltic lava lakes and lava flows in the first 4 hours from the emission. It is generally assumed that changes in mineral assemblage, crystal content and rheological properties of lava flows occur instantaneously with changes in temperature. However, transient crystallisation processes and delay of crystal nucleation occur in isothermal conditions, meaning that crystallisation and melt viscosity can change with time^3,53^. Crystal content controls the rheology of lava flows and our results show that crystallisation kinetics may slow the crystallisation process, promoting lower viscosities and longer flow lengths. An archetype for a very fast-moving lava flow was produced during the 1981 eruption of Mount Etna. This eruptive episode was characterised by the emplacement of a ~6 km-long lava flow in about 3 hours (17 March 1981)^25^. Our isothermal experiment ET1170 at 1170 °C shows a negligible amount of pyroxene crystals (<0.001) and ~0.10 of oxides after 3 hours, whereas, considering the same duration in isothermal experiment ET1150 at 1150 °C the crystal fraction of Fe-Mg oxides is 0.16 and that of pyroxene is 0.05. From a rheological point of view, the spherical shapes of Fe-Mg oxides have a negligible effect on the viscosity of lava flows, whereas the elongated shapes of pyroxene crystals could affect lava viscosity^3^; however, in agreement with ref.^3^, we suggest that the low pyroxene crystal fraction in the first 3 hours was not enough to induce a dramatic viscosity change. In ref.^3^, Vona *et al*. performed isothermal experiments, studying the viscosity evolution of Etna basalt with time. However, through their *ex situ* experiments it is not possible to quantify the amount of crystals and which phases are produced during the first hours of the experiments, and therefore, 3D information on the crystal textural evolution in real time cannot be obtained. Our 3D real-time experiments illustrate that a small amount of pyroxene crystals formed during the first 3 hours, implying that these crystals did not increase lava viscosity significantly during its emplacement. This finding explains the reason why lava can flow for long distances in a short time^25^. The novelty of our approach is that we can quantify in 3D the evolution of crystallisation in real time, showing exactly the onset of crystallisation of each mineral phase. This aspect allows us to investigate the textural evolution of basaltic melts at the beginning of the solidification process when the magma is still able to move and flow, providing a fundamental improvement in comparison to *ex situ* crystallisation and rheological experiments. This study provides the first *in situ* time-dependent measurements of 3D disequilibrium crystal nucleation and growth in a natural magma and opens the possibility to study volcanic melts under realistic conditions for volcanic systems. The *in situ* 4D technique provides orders of magnitude increase in the number of experimental runs that can be conducted compared with traditional *ex situ* experiments, opening a new frontier for experimental petrology and volcanology. Our results provide a fundamental improvement of our knowledge of crystallisation in basaltic magmas, with implications for the rheological behaviour of basaltic magma, which is strongly dependent on crystal content and crystal morphology, at near-vent conditions, relevant to the flow of lava and the ascent of explosive magma in the shallow conduit. As such, our results should be used in models of lava flow dynamics and emplacement, and of magma ascent in the shallow conduit, to improve their capacity to forecast the evolution of volcanic eruptions, assess volcanic hazard and reduce the related volcanic risk.

## Methods

### Choice of starting material, starting material preparation and composition, pyroxene composition

The starting material used for our cooling-driven crystallisation experiments consists of volcanic products from the lower vents of the 2001 Mt. Etna eruption^54–56^. In our experiments, we do not attempt to reproduce faithfully the petrological and textural characteristics of the natural erupted products: rather, we use the eruption and its products as a case study to investigate crystallisation processes in shallow basaltic systems. It is however worth highlighting that our experimentally produced pyroxene crystals resemble both compositionally (see Supplementary Fig. S1 and Supplementary Table S3) and texturally (ubiquitous presence of crystal aggregates, Fig. 2a,b in ref.^55^) those found in natural samples. We chose material from the 2001 eruption because i) in terms of style and intensity, this is one of the most representative eruptions that Mt. Etna has produced in the last couple of decades, and can therefore be considered an archetype eruption for scientists investigating processes in basaltic systems; ii) this is one of the most well monitored and studied eruptions at this volcano, and there are numerous studies in the literature addressing the eruption chronology and dynamics^54,56–58^, its geophysical^59^ and geochemical and petrological features^55,60^, and numerical modelling of conduit magma ascent^18^.

The anhydrous, glassy starting material was obtained by melting crushed rock samples in a 100 ml large, thin-walled, Pt crucible. Melting was performed in a Nabertherm® MoSi_2_ box furnace at 1400 °C in air and at atmospheric pressure by adding the crushed sample to the crucible every 15 minutes. Once the crucible was completely filled, the melt was left in the furnace for about four hours to allow the melt to fully degas. The melt was then quenched in air to glass by pouring it onto a steel plate, and was crushed and melted a second time. Finally, glassy cylinders 3 mm in diameter and 4 mm in length were drilled from the synthesized glass for synchrotron X-ray microtomography experiments.

The chemical composition of the glassy starting material and of pyroxene crystals have been analysed with a Jeol JXA 8530 F microprobe in the facilities of the School of Earth and Environmental, Sciences, University of Manchester, UK, and are reported in Supplementary Table S1, Supplementary Table S3 and Supplementary Fig. S1. Analyses were performed using a 15 kV accelerating voltage, 10 nA beam current and beam size of 10 μm. Standards used for calibration were albite for Na, periclase for Mg, corundum for Al, fayalite for Fe, tephroite for Mn, apatite for P, sanidine for K, wollastonite for Ca and Si and rutile for Ti. Sodium and potassium were measured first to minimize loss owing to volatilisation.

### Synchrotron X-ray microtomography experiments of 4D crystallisation in the 2001 Mt. Etna basalt

Cooling-driven crystallisation experiments are defined by the temperature decrement imposed during cooling^9^: either one large drop in T (named single-step cooling or SSC) or a continuous lowering of T (named continuous cooling or CC). Our experiments ET1150 and ET1170 belong to the former type of crystallisation experiments (Supplementary Table S2). All experiments were performed at Diamond Light Source beamline I12. Small cylindrical chips from the glassy starting material described above were put in an alumina sample holder and were heated to 1250 °C and then equilibrated at this temperature for 30 minutes before cooling. For these experiments, we used the high-temperature resistance Alice furnace^14^, which was commissioned and successfully used up to 1460 °C on I12, with controlled cooling at 0.05 °C/sec to 0.5 °C/sec. Crystallisation was induced by isobarically decreasing temperature from 1250 °C to 1170 °C or 1150 °C, and then holding at the final temperature for a dwell time of 4 h. Each experiment lasted about 6 ½ h in total. Both experiments were conducted in phase-contrast mode with a detector-sample distance of 2300 mm, using monochromatic 53 keV light at a temporal resolution of 3 min per scan and a pixel size of 3.2 μm. The detector was a high-resolution imaging PCO.edge camera with optical module 3, corresponding to a field of view of 8.0 mm × 7.0 mm. Because we wanted to check the conditions of the starting material both before and after the experiments, we acquired one scan of the sample in static mode at the beginning and end of each experiment. Continuous scanning started before the end of sample melting/equilibration, covered cooling and lasted for the entire duration of the dwell time. In each scan, 1800 tomographic projections were acquired by the detector with equiangular steps over a full rotation angle of 180° and an exposure time/projection of 0.05 s. These experimental conditions were sufficient to capture pyroxene nucleation and growth and oxide crystallisation processes and their textural evolution in 3D through time.

### Tomographic data processing and quantitative analysis

Tomographic projections were reconstructed into 2D slices by using Diamond I12 in-house python codes. The pre-processing pipeline includes centre of rotation calculation, zinger removal, blob removal, regularisation-based ring removal, then the GRIDEC algorithm is used for reconstruction (http://confluence.diamond.ac.uk/display/I12Tech/Reconstruction+scripts+for+time+series+tomography, and refs^61,62^). The resulting 2D reconstructed tomographic slices were converted to 8-bit raw format and stacked into the freeware ImageJ software^63^ to produce 3D digital volumes where the isotropic voxel size has an edge length of 3.2 μm. 3D visualisation (volume rendering) of the reconstructed volumes was obtained with the commercial software VGStudio 3.0 (Volume Graphics), which allowed us to make qualitative textural observations on the evolution of pyroxene and oxide nucleation and growth kinetics with time (Fig. 1, Supplementary Movie S1, Supplementary Movie S2). While the whole scan time series consisting of 80 frames and covering the dwell time was visualised carefully for qualitative textural investigation, quantitative image analysis was performed on 16 frames with a time window of 15 minutes between the selected frames. Reconstructed volumes of SSC experiment ET1150 were then cropped with ImageJ to select a volume of interest (VOI) for quantitative image analysis of pyroxene and oxide crystals. The VOI was selected carefully in order to i) discard the original imaged volume edges, which in the reconstructed volumes included the experimental apparatus, and ii) specifically avoid the two entrained air bubbles at the bottom and top of the sample holder, as well as small bubbles next to the sample holder walls that would have complicated image segmentation owing to their highly phase-contrasted walls, and iii) avoid regions where thermal gradients were the highest, i.e. the top and bottom of the sample holder. The VOI in each frame therefore corresponds to the central part of the sample (Supplementary Fig. S2), which represents the furnace hotspot and the largest available melt volume that remains unaffected by surface effects. In experiment ET1170 pyroxene crystals nucleated in negligible numbers (crystal volume fraction <0.001) and only at the bottom of the sample holder at the melt/sample holder interface, together with oxide crystals (0.11 ± 0.01). In this experiment we analysed pyroxene and oxide textures only qualitatively, and used this information to provide pyroxene and oxide nucleation delay times for comparison with experiment ET1150.

We performed image processing of the selected VOIs following the protocol described in ref.^64^ (Supplementary Table S4). Segmentation is the process that allows separation of objects from the background to obtain binary volumes containing only the feature of interest. After using the brightness-contrast function in ImageJ to increase the intensity of crystals and decrease that of the matrix, segmentation of pyroxene crystals was operated in the 3D domain with the Pore3D software library^65^ by using manual bi-level greyscale thresholding based on the greyscale histogram of the selected VOIs and visual inspection of the slices in different directions. This allowed high sensitivity to the presence of noise and artefacts and proved favourable for our purpose compared with automatic thresholding algorithms. To avoid biasing from texturally complicated microtomographic images, pre- and post-segmentation smoothing filters were required to both ease and refine the segmentation procedure (Supplementary Table S4, and refs^66–68^). The 3D bilateral filter in Pore3D was applied to smooth the greyscale input images prior to segmentation, while preserving object edges^69^. Because oxide crystals have a near-identical greyscale contrast to pyroxene crystals in our VOIs, a significant fraction of oxides were segmented together with pyroxenes in the same binary VOIs. To obtain pyroxene crystal segmentation, we removed the segmented oxides from the volumes using a combination of post-segmentation image processing steps in both ImageJ and Pore3D. This consisted in applying a series of consecutive binary operations in ImageJ such as open, erosion, remove outliers and dilate on both sagittal and coronal planes, which removed oxides and only a negligible part of the smallest pyroxene crystals, as well as using the 3D minimum volume filter (MVF) in Pore3D, in the range 500–1500 pixel^3^ (corresponding to a volume of 25^3^ μm^3^ and 37^3^ μm^3^). An example of the procedure is illustrated in Supplementary Movie S2 and Supplementary Movie S3. Here, a volume rendering of frame 40 after manual greyscale thresholding and displaying both pyroxene and oxide crystals (Supplementary Movie S2) is compared with a volume rendering of the same frame containing only pyroxenes after the post-segmentation image processing protocol described above (Supplementary Movie S3). The whole image processing protocol, including segmentation, pre- and post-segmentation processing, lasted up to 3–4 hours per VOI. The resulting segmented pyroxene crystals were counted in each VOI and their volumes computed with the blob analysis in Pore3D^70^. Pyroxene crystals numbers and individual volumes were used to obtain the textural and kinetic parameters reported in Table 1, and Figs 2 and 3. Oxide volume fractions were computed using the plugin BoneJ in ImageJ, while their average crystallisation rate was obtained following the same equation reported in the following for pyroxenes. Specifically, for each frame *n* acquired at the time *t*^*(n)*^, given the volume of pyroxene *V*_*px*_*(t*^*(n)*^) and the number of pyroxene crystals *N*_*px*_*(t*^*(n*)^*)*, we computed:

4 $$\varphi ({t}^{(n)})=\frac{{V}_{px}({t}^{(n)})}{VOI};$$

5 $${N}_{v}({t}^{(n)})=\frac{{N}_{px}({t}^{(n)})}{VOI};$$

6 $${I}_{v}({t}^{(n)})=\frac{{N}_{v}({t}^{(n)})}{{t}^{(n)}};$$

7 $${Y}_{v}({t}^{(n)})=\frac{\varphi ({t}^{(n)})}{{t}^{(n)}};$$

8 $${Y}_{iv}({t}^{(n+1)})=\frac{\varphi ({t}^{(n+1)})-\varphi ({t}^{(n)})}{{t}^{(n+1)}-{t}^{(n)}};$$

where *ϕ* is the crystal volume fraction, *Nv* is the crystal number density, *Iv* is the average crystal nucleation rate, *Yv* is the average crystallisation rate, and *Yiv* is the instantaneous crystallisation rate. The uncertainty associated with image processing and analysis was calculated at 6% for *ϕ* and 7% for *Nv*, *Ivt*, and *Yvt*, and the standard deviation of the mean value is reported for each parameter in Tables 1 and 2.

### Data availability

The data that support the findings of this study are available within the article, its Supplementary Information files and from the corresponding author upon request.

## Electronic supplementary material

Supplementary material

Supplementary TableS3

Supplementary MovieS1

Supplementary MovieS2

Supplementary MovieS3

## References

[1] Rust, A.C. & Cashman, K.V. Permeability of vesicular silicic magma: inertial and hysteresis effects. *Earth Planet. Sci. Lett*. **228**, 93–107.

[2] Mader HM, Llewellin EW, Mueller SP (2013). The rheology of two-phase magmas: A review and analysis. J. Volcanol. Geotherm. Res..

[3] Vona A, Romano C, Dingwell DB, Giordano D (2011). The rheology of crystal-bearing magmas. Geoch. Cosmoch. Acta.

[4] Pistone M (2012). Deformation experiments of bubble- and crystal-bearing magmas: rheological and microstructural analysis. J. Geophys. Res..

[5] Polacci M (2014). Permeability measurements of Campi Flegrei pyroclastic products: An example from the Campanian Ignimbrite and Monte Nuovo eruptions. J. Volcanol. Geotherm. Res..

[6] Vona A (2017). The complex rheology of megacryst-rich magmas: The case of the mugearitic “cicirara” lavas of Mt. Etna volcano. Chem. Geol..

[7] Brugger CR, Hammer JE (2010). Crystallization kinetics in continuous decompression experiments: implications for interpreting natural magma ascent processes. J. Petrol..

[8] Arzilli F, Carroll MR (2013). Crystallization kinetics of alkali feldspars in cooling and decompression-induced crystallization experiments in trachytic melt. Contrib. Mineral. Petrol..

[9] Shea T, Hammer JE (2013). Kinetics of cooling- and decompression-induced crystallization in hydrous mafic-intermediate magmas. J. Volcanol. Geotherm. Res..

[10] Arzilli F (2015). Plagioclase nucleation and growth kinetics in a hydrous basaltic melt by decompression experiments. Contrib. Mineral. Petrol..

[11] Sunagawa, I. Crystals. Growth, Morphology and Perfection. Cambridge University Press, 295 pp (2005).

[12] Schiavi F, Walte N, Keppler H (2009). First *in situ* observation of crystallization processes in a basaltic-andesitic melt with the moissanite cell. Geology.

[13] Karagadde S (2015). Transgranular liquation cracking of grains in the semi-solid state. Nat. Commun..

[14] Azeem MA (2017). Revealing dendritic pattern formation in Ni, Fe and Co alloys using synchrotron tomography. Acta Mater..

[15] Barclay J, Carmichael ISE (2004). A hornblende basalt from western Mexico: water-saturated phase relations constrain a pressure–temperature window of eruptibility. J. Petrol..

[16] Vona A, Romano C (2013). The effect of undercooling and deformation on the crystallization kinetics of Stromboli and Etna basalts. Contrib. Mineral. Petrol..

[17] Agostini C, Fortunati A, Arzilli F, Landi P, Carroll MR (2013). Kinetics of crystal evolution as a probe to magmatism atStromboli (Aeolian Archipelago, Italy). Geoch. Cosmoch. Acta.

[18] La Spina G, Burton MR, de’Michieli Vitturi M, Arzilli F (2016). Role of syn-eruptive plagioclase disequilibrium crystallization in basaltic magma ascent dynamics. Nat. Commun..

[19] Pichavant M (2013). Generation of CO_2_-rich melts during basalt magma ascent and degassing. Contrib. Mineral. Petrol..

[20] Lloyd AS (2014). NanoSIMS results from olivine-hosted melt embayments: Magma ascent rate during explosive basaltic eruptions. J. Volcanol. Geotherm. Res..

[21] Le Gall N, Pichavant M (2016). Experimental simulation of bubble nucleation and magma ascent in basaltic systems: Implications for Stromboli volcano. Am. Mineral..

[22] Pupier E, Duchese S, Toplis MJ (2008). Experimental quantification of plagioclase crystal size distribution during cooling of a basaltic liquid. Contrib. Mineral. Petrol..

[23] Zieg MJ, Lofgren GE (2006). An experimental investigation of texture evolution during continuous cooling. J. Volcanol. Geotherm. Res..

[24] Ni H (2014). *In situ* observation of crystal growth in a basaltic melt and the development of crystal size distributions in igneous rocks. Contrib. Mineral. Petrol..

[25] Coltelli M, Marsella M, Proietti C, Scifoni S (2012). The case of 1981 eruption of Mount Etna: An example of very fast moving lava flows. Geochem. Geophys. Geosyst..

[26] Cashman KV, Thornber C, Kauahikaua JP (1999). Cooling and crystallization of lava in open channels, and the transition of Pa¯hoehoe Lava to ‘A’a. Bull. Volcanol..

[27] Witter JB, Harris AJL (2007). Field measurements of heat loss from skylights and lava tube systems. J. Geophys. Res..

[28] Ghiorso MS, Sack RO (1995). Chemical mass transfer in magmatic processes IV. A revised and internally consistent thermodynamic model for the interpolation and extrapolation of liquid-solid equilibria in magmatic systems at elevated temperatures and pressures. Contrib. Mineral. Petrol..

[29] Asimow PD, Ghiorso MS (1998). Algorithmic modifications extending MELTS to calculate subsolidus phase relations. Am. Mineral..

[30] Orlando A, D’Orazio M, Armienti P, Borrini D (2008). Experimental determination of plagioclase and clinopyroxene crystal growth rates in an anhydrous trachybasalt from Mt Etna (Italy). Eur. J. Mineral..

[31] Kelton, K. F. & Greer, A. L. Nucleation in Condensed Matter: Applications in Materials and Biology. *Elsevier*, Amsterdam, ISBN: 978-0-08-042147-6 (2010).

[32] Couch S (2003). Experimental investigation of crystallization kinetics in a haplogranite system. Am. Mineral..

[33] Donaldson CH (1979). An experimental investigation of the delay in nucleation of olivine in Mafic Magmas. Contrib. Mineral. Petrol..

[34] Hammer JE (2004). Crystal nucleation in hydrous rhyolite: Experimental data applied to classical theory. Am. Mineral..

[35] Hammer JE, Sharp TG, Wessel P (2010). Heterogeneous nucleation and epitaxial crystal growth of magmatic minerals. Geology.

[36] Arzilli F (2015). Near-liquidus growth of feldspar spherulites in trachytic melts: 3D morphologies and implications in crystallization mechanisms. Lithos.

[37] Giordano D, Russell JK, Dingwell DB (2008). Viscosity of magmatic liquids: a model. Earth Planet. Sci. Lett..

[38] Hammer JE (2008). Experimental studies of the kinetics and energetics of magma crystallization. Rev. Mineral. Geochem..

[39] Davis MJ, Ihinger PD (2008). Heterogeneous crystal nucleation on bubbles in silicate melt. Am. Mineral..

[40] Gimeno D (2003). Devitrification of natural rhyolitic obsidian glasses: petrographic and microstructural study (SEM+EDS) of recent (Lipari island) and ancient (Sarrabus, SE Sardinia) samples. J. Non-Crys. Solids.

[41] Clay PL (2012). Textural characterization, major and volatile element quantification and Ar–Ar systematics of spherulites in the Rocche Rosse obsidian flow, Lipari, Aeolian Islands: a temperature continuum growth model. Contrib. Mineral. Petrol..

[42] Polacci M (2017). From magma ascent to ash generation: investigating volcanic conduit processes by integrating experiments, numerical modeling, and observations. Ann. Geophys..

[43] Gránásy L, Pusztai T, Borzsonyi T, Warren JA, Douglas JF (2004). A general mechanism of polycrystalline growth. Nat. Mater..

[44] Gránásy L, Pusztai T, Tegze G, Warren JA, Douglas JF (2005). Growth and form of spherulites. Phys. Rev. E.

[45] Smith CS (1964). Some elementary principles of polycrystalline microstructure. Metal. Rev..

[46] Ikeda S, Toriumi M, Yoshida H, Shimizu I (2002). Experimental study of the textural development of igneous rocks in the late stage of crystallization: the importance of interfacial energies under non-equilibrium conditions. Contrib. Mineral. Petrol..

[47] Marsh BD (1988). Crystal size distribution (CSD) in rocks and the kinetics and dynamics of crystallization. I. Theory. Contrib. Mineral. Petrol..

[48] Cashman KV, Marsh BD (1988). Crystal size distribution (CSD) in rocks and the kinetics and dynamics of crystallization II: Makaopuhi lava lake. Contrib. Mineral. Petrol..

[49] Marsh BD (1998). On the interpretation of crystal size distributions in magmatic systems. J. Petrol..

[50] Armienti P, Pareschi MT, Innocenti F, Pompilio M (1994). Effects of magma storage and ascent on the kinetics of crystal growth. Contrib. Mineral. Petrol..

[51] La Spina G, Burton M (2015). de’ Michieli Vitturi, M. Temperature evolution during magma ascent in basaltic effusive eruptions: a numerical application to Stromboli volcano. Earth Planet. Sci. Lett..

[52] Melnik O, Sparks RSJ (2005). Control on conduit magma flows dynamics during lava dome building eruptions. J. Geophys. Res..

[53] Kolzenburg S, Giordano D, Cimarelli C, Dingwell DB (2016). *In situ* thermal characterization of cooling/crystallizing lavas during rheology measurements and implications for lava flow emplacement. Geoch. Cosm. Acta.

[54] Behncke B, Neri M (2003). The July-August 2001 eruption of Mt. Etna (Sicily). B. Volcanol..

[55] Corsaro RA, Miraglia L, Pompilio M (2007). Petrologic evidence of a complex plumbing system feeding the July-August 2001 eruption of Mt. Etna, Sicily, Italy. B. Volcanol..

[56] Calvari S (2001). Multidisciplinary approach yields insights into Mt. Etna 2001 eruption. Eos Trans. AGU.

[57] Taddeucci J, Pompilio M, Scarlato P (2005). Conduit processes during the July-August 2001 explosive activity of Mt. Etna (Italy): inferences from glass chemistry and crystal size distribution of ash particles. J. Volcanol. Geotherm. Res..

[58] Viccaro M, Ferlito C, Cortesogno L, Cristofolini R, Gaggero L (2006). Magma mixing during the 2001 event at Mount Etna (Italy): effects on the eruptive dynamics. J. Volcanol. Geotherm. Res..

[59] Bonforte A, Gambino S, Neri M (2009). Intrusion of eccentric dikes: the case of the 2001 eruption and its role in the dynamics of Mt. Etna volcano. Tectonophysics.

[60] Métrich N, Allard P, Spilliaert N, Andronico D, Burton M (2004). 2001 flank eruption of the alkali- and volatile-rich primitive basalt responsible for Mount Etna’s evolution in the last three decades. Earth Planet. Sci. Lett..

[61] Vo NT, Drakopoulos M, Atwood RC, Reinhard C (2014). Reliable method for calculating the center of rotation in parallel-beam tomography. Opt. Express.

[62] Vo NT, Atwood RC, Drakopoulos M (2015). Radial lens distortion correction with sub-pixel accuracy for X-ray micro-tomography. Opt. Express.

[63] Abramoff MD, Magelhaes PJ, Ram SJ (2004). Image processing with ImageJ. Biophot. Int..

[64] Arzilli F (2016). A novel protocol for resolving feldspar crystals in synchrotron X-ray microtomographic images of crystallized natural magmas and synthetic analogues. Am. Mineral..

[65] Brun F (2010). Pore3D: A software library for quantitative analysis of porous media. Nucl. Inst. Meth. Phys. Res. A.

[66] Polacci M, Mancini L, Baker DR (2010). The contribution of synchrotron X-ray computed microtomography to understanding volcanic processes. J. Synchrotron. Rad..

[67] Zandomeneghi D (2010). Quantitative analysis of X-ray microtomography images of geomaterials: Application to volcanic rock. Geosphere.

[68] Polacci M, Baker DR, LaRue A, Mancini L (2012). Degassing behaviour of vesiculated basaltic magmas: an example from Ambrym volcano, Vanuatu Arc. J. Volcanol. Geotherm. Res..

[69] Tomasi, C. & Manduchi, R. Bilateral filtering for gray and color images. *Proceedings of the 1998 IEEE International Conference on Computer Vision*, Bombay, India, 839–846 (1998).

[70] Hu Q, Qian G, Nowinski WL (2005). Fast connected-component labeling in three-dimensional binary images based on iterative recursion. Comput. Vis. Image Understand..

