# Supplementary material for: Crystallisation in basaltic magmas revealed via in situ 4D synchrotron X-ray microtomography
Source: Sci Rep. 2018 May 30;8:8377. doi: 10.1038/s41598-018-26644-6 (PMC5976632; doi:10.1038/s41598-018-26644-6)
Supplement: Supplementary file 1 — Supplementary material [file 41598_2018_26644_MOESM1_ESM.pdf]

Crystallisation in basaltic magmas revealed via *in situ* 4D synchrotron X-ray microtomography

M. Polacci[1*], F. Arzilli[1], G. La Spina[1], N. Le Gall[2, a], B. Cai[2, a,b] , M. E. Hartley[1], D. Di Genova[3], N. T. Vo[4], S. Nonni[2, a], R. C. Atwood[4], E. W. Llewellin[5], P. D. Lee[2, a] and M. R. Burton[1]

1) School of Earth and Environmental Sciences, University of Manchester, Manchester M13 9PL, UK

2) School of Materials, University of Manchester, Manchester M13 9PL, UK

a) Research Complex at Harwell, Harwell Campus, OX 11 0FA, UK

b) Now at School of Metallurgy and Materials, University of Birmingham, Edgbaston, Birmingham, B15 2TT, UK

3) School of Earth Sciences, University of Bristol, Bristol BS8 1RJ, UK

4) Diamond Light Source, Harwell Science and Innovation Campus, Didcot OX11 0DE, UK

5) Dept. Earth Sciences, Durham University, Durham DH1 3LE, UK

*Corresponding author at: School of Earth and Environmental Sciences, University of Manchester, Manchester M13 9PL, UK, email: margherita.polacci@manchester.ac.uk

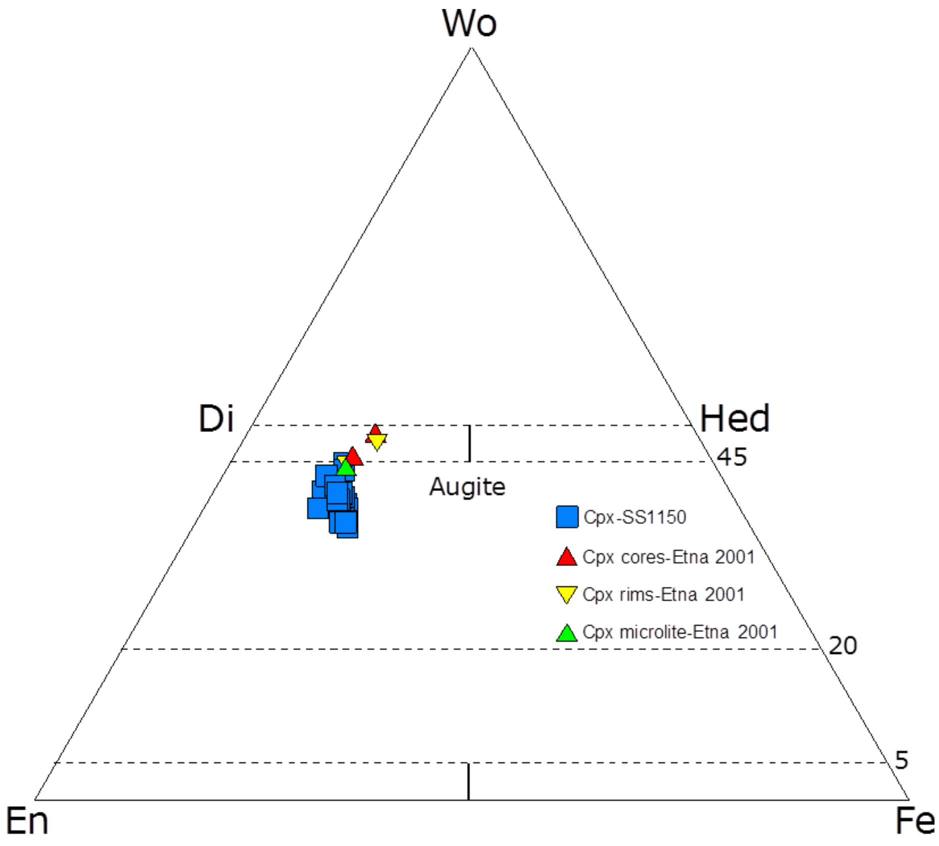

Supplementary Figure S1 Cpx ternary diagram with compositions of pyroxenes from single-step cooling experiment ET1150 plotted together with compositions of natural pyroxenes from the 2001 Mt. Etna eruption (Corsaro et al. 2007).

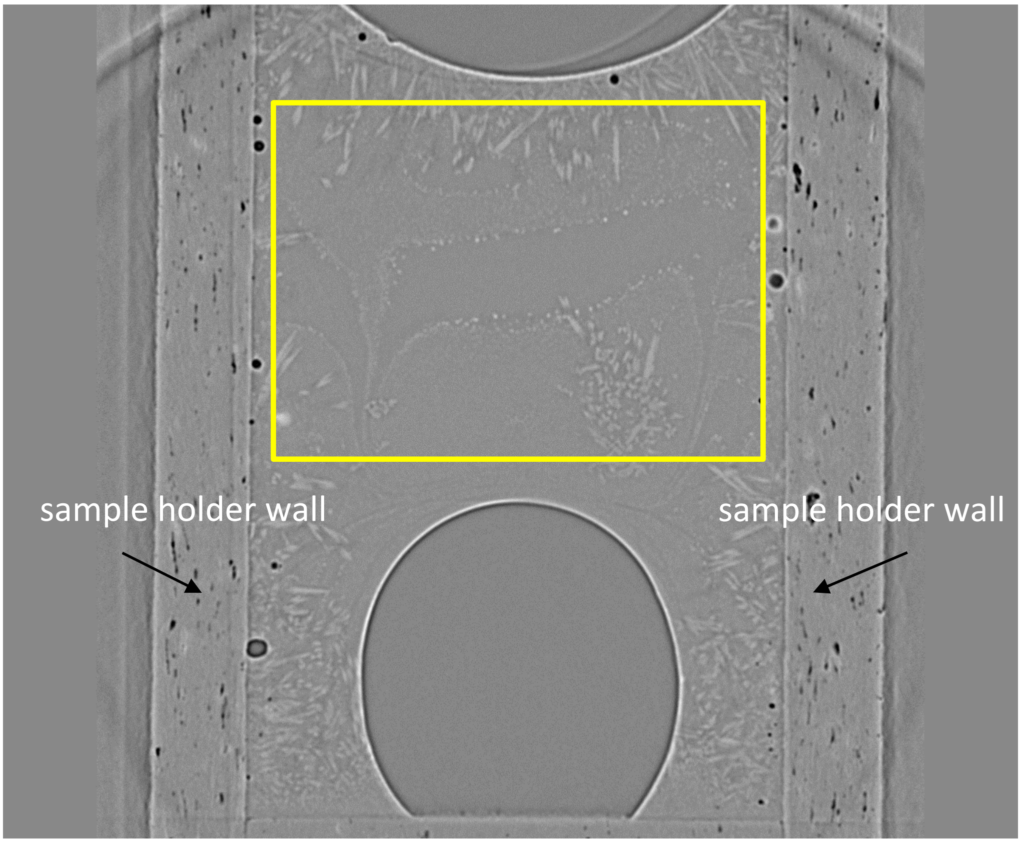

Supplementary Figure S2 Coronal view of 2D microtomographic slice from frame 40 illustrating sample holder walls, the sample in the sample holder and the VOI (yellow inset) selected for quantitative image analysis. Horizontal field of view is 5 mm.

Table S1. Chemical composition of the starting material.

Oxide	Bulk composition wt.%	St. dev.
SiO_2	48.40	0.16
TiO_2	1.79	0.07
Al_2O_3	16.22	0.50
FeO	10.66	0.30
MnO	0.20	0.03
MgO	6.20	0.32
CaO	10.66	0.19
Na_2O	3.41	0.20
K_2O	1.90	0.07
P_2O_5	0.54	0.04
Total	99.85	

note: FeO* = total iron as FeO.

Table S2 Experimental conditions for single-step cooling experiments

Experiment	Heating time to Ti (min)	Ti (°C)	Tf (°C)	Melting time at Ti (min)	Cooling rate to Tf (°C/sec)	Cooling time to Tf (min)	Dwell time at Tf (min)	Furnace cooling to room T (min)	Total time (h)
ET1150	~ 60	1250	1150	30	0.4	~5	240	~50	~6.5
ET1170	~ 60	1250	1170	30	0.4	~5	240	~50	~6.5

Note: Ti and Tf indicate initial and final experimental temperature

Table S4 Summary of X-ray microtomography acquisition conditions and image processing details of the volumes of interest (VOIs) for experiment ET1150

Instrument	Voxel size μm^3	Original imaged volumes (pixels) (mm^3)	Analysed VOIs (mm^3)	Pre-segmentation	Segmentation	Post-segmentation
DLS I12	3.2x3.2x3.2	1683x1683x1383 (128.4)	(8.2037)	bilateral filter (Pore3D)	greyscale thresholding (Pore3D)	open, remove outliers, erode, dilate (ImageJ)+MVF (Pore3D)

Supplementary Table S3 Individual excel file with data on microprobe analysis of pyroxene crystals

Caption Supplementary Movie S1 Volume rendering movie of the entire pyroxene crystallisation process in single-step cooling experiment ET1150. Vertical field of view is 2 mm.

Caption Supplementary Movie S2 Volume rendering movie of frame 40 VOI after greyscale thresholding illustrating both pyroxene and oxide crystals. Vertical field of view is 2 mm.

Caption Supplementary Movie S3 Volume rendering movie of frame 40 VOI after entire post-segmentation image processing protocol described in this study and illustrating only segmented pyroxene crystals. Vertical field of view is 2 mm.
